# Model predictive control for steady-state performance in integrated continuous bioprocesses

**DOI:** 10.1007/s00449-022-02759-z

**Published:** 2022-08-02

**Authors:** Magdalena Pappenreiter, Sebastian Döbele, Gerald Striedner, Alois Jungbauer, Bernhard Sissolak

**Affiliations:** 1 Innovation Management, Bilfinger Life Science GmbH, Salzburg, Austria; 2 Department of Biotechnology, Institute of Bioprocess Science and Engineering, University of Natural Resources and Life Sciences, Vienna, Austria

**Keywords:** Perfusion process, Bleed, Feedback control, Single prediction control, Antibody

## Abstract

Perfusion bioreactors are commonly used for the continuous production of monoclonal antibodies (mAb). One potential benefit of continuous bioprocessing is the ability to operate under steady-state conditions for an extended process time. However, the process performance is often limited by the feedback control of feed, harvest, and bleed flow rates. If the future behavior of a bioprocess can be adequately described, predictive control can reduce set point deviations and thereby maximize process stability. In this study, we investigated the predictive control of biomass in a perfusion bioreactor integrated to a non-chromatographic capture step, in a series of Monte-Carlo simulations. A simple algorithm was developed to estimate the current and predict the future viable cell concentrations (VCC) of the bioprocess. This feature enabled the single prediction controller (SPC) to compensate for process variations that would normally be transported to adjacent units in integrated continuous bioprocesses (ICB). Use of this SPC strategy significantly reduced biomass, product concentration, and harvest flow variability and stabilized the operation over long periods of time compared to simulations using feedback control strategies. Additionally, we demonstrated the possibility of maximizing product yields simply by adjusting perfusion control strategies. This method could be used to prevent savings in total product losses of 4.5–10% over 30 days of protein production.

## Introduction

Advancements of perfusion culture have enabled the realization of integrated continuous and intensified bioprocesses [1–6]. In perfusion culture, a cell retention device retains cells in the bioreactor, while medium is continuously fed and product constantly harvested [7–10]. This enables continuous cell cultivation, with achievement of a high titer and consistent product quality [11, 12]. Perfusion cultures are particularly attractive for multi-production sites, as they necessitate lower capital investment costs, and enable the use of smaller equipment and flexible ways of working [13–15]. Systemic development of media is lowering its costs, which will further reduce the concerns regarding the cost of goods (COG) [16, 17].

The challenge of perfusion culture is to maintain a constant biomass over a long period of time because cell growth cannot be fully arrested. Continuous perfusion cultures are complex systems with multiple inputs and outputs, and therefore require appropriate process control. However, most experimental applications and reported studies are based on simple models and follow single input and output strategies (SISO), like feedback control algorithms [18–20]. Closed-loop systems are designed to achieve and maintain the desired output conditions through comparison with the actual state. Such control strategies are sometimes limited in their use for stable long-term control as they can introduce disturbances into other control loops. This is especially undesired in the context of process integration of perfusion with continuous downstream processing.

Simulation studies have successfully demonstrated different approaches—based on the principle of model predictive control (MPC)—to address the multivariable nature of such processes [21]. Moreover, the implementation of process analytical technology (PAT) in an automation framework develops a well-controlled process using sensor feedback signals that enable model predictive control strategies at an advanced level [14, 22]. Use of the MPC control scheme, in which a mathematical model is used to predict its future trajectory, can minimize the generated error signal. This has been addressed, for example, by novel control schemes in perfusion and continuous systems [23, 24] by supporting continuous bio production in downstream purifications [25] or by controlling product quality attributes in antibody manufacture [26, 27]. A so-called single prediction controller (SPC) anticipates the future behavior based on only one prediction of the system output. Consequently, it improves system performance through reduction of disturbances and fluctuations [28].

The present study extends the previous analyses of integrated continuous bioprocesses [1, 5, 11, 22, 29–33] with particular focus on simulating the error propagation from perfusion control concepts to downstream units. Papathanasiou et al. successfully overcame this issue with the implementation of a multiparametric model predictive control in a semi-continuous purification process to overcome this issue. They were able to efficiently deal with measured disturbances originating from the upstream process, which, in this case, could not be controlled by the user [25]. However, this method can have limitations, especially when a truly continuous mass flow is applied and the unit is directly connected to the upstream unit. We have developed a different solution and exemplified this with a simulated showcase for commercial antibody manufacture. Different strategies were developed to control perfusion processes under constant and varying harvest flow, using a single prediction control strategy. The strategies were applied in Monte-Carlo simulation studies and compared with feedback control.

In the field of continuous biomanufacturing, the proposed approach makes several novel contributions: (i) it defines the requirements of global process control strategies for integrated continuous bioprocesses compared to single perfusion processes; (ii) it provides a straightforward solution to stably controlled perfusion systems that are directly connected to downstream unit operations (without mass flow interruptions); and (iii) it thereby minimizes the control burden in the downstream process while being sensitive to product concentration and harvest flow variations. We propose that a predictive control structure can efficiently manipulate the bleed rate, which is particularly important for efficient operation in a perfusion process as it influences other parameters. Moreover, our proposed framework is simple to implement and is not limited to a specific process, but may be extensively applied in integrated continuous biomanufacturing.

## Methods

### Perfusion process model for steady-state conditions

To study the error propagation of a perfusion system to subsequent downstream units, we developed a simplified process model solely based on the mass balance equation and the assumption that the volume exchange per day (VVD) remains constant.

In a perfusion system the in- and output flow rates should equal zero for maintaining a constant bioreactor hold-up volume using Eq. (1):

1 $$\frac{{\dot{V}}_{P}}{{V}_{r}}-\frac{{\dot{V}}_{B}}{{V}_{r}}-\frac{{\dot{V}}_{H}}{{V}_{r}}=0,$$

with ${\dot{V}}_{{P}}$, ${\dot{V}}_{{B}}$ and ${\dot{V}}_{{H}}$ depicting the volumetric flowrate of the perfusion feed, the bleed and the harvest, respectively, and with

2 $$P=\frac{{\dot{V}}_{P}}{{V}_{r}}; \; B= \frac{{\dot{V}}_{B}}{{V}_{r}}; \; H=\frac{{\dot{V}}_{H}}{{V}_{r}}.$$

Equation (2) can be further simplified to

3 $$P =B+H,$$

which is the major boundary condition for this process model. The perfusion rate *P* is the sum of the bleed rate *B* and the harvest rate *H*. The perfusion rate at which new medium is supplied to the vessel is usually expressed as specific rate (*P*) in volumes of medium per vessel volume per day and *V*_in_ represents the media flow rate per day (L_media_ d^−1^).

4 $$P= \frac{{V}_{\mathrm{in}}}{{V}_{r}}$$

Conclusively, the change of biomass (*X*) over time in a perfusion system is described as the sum of grown and bleeded out cells. Hence,

5 $$\frac{\mathrm{d}X}{\mathrm{d}t}=\mu \cdot X -B\cdot X,$$

with *µ* depicting the growth rate. Solving the integration results in

6 $${\int }_{{X}_{0}}^{{X}_{i}}\frac{1}{X}\mathrm{d}X={\int }_{0}^{t}\mu \mathrm{d}t- {\int }_{0}^{t}B\mathrm{d}t,$$

7 $$\mathrm{ln}\frac{{X}_{i}}{{X}_{0}}=\left(\mu -B\right)\Delta t,$$

8 $${X}_{i}={X}_{0}\cdot {e}^{\left(\mu -B\right)\Delta t}.$$

**Fig. 1:**
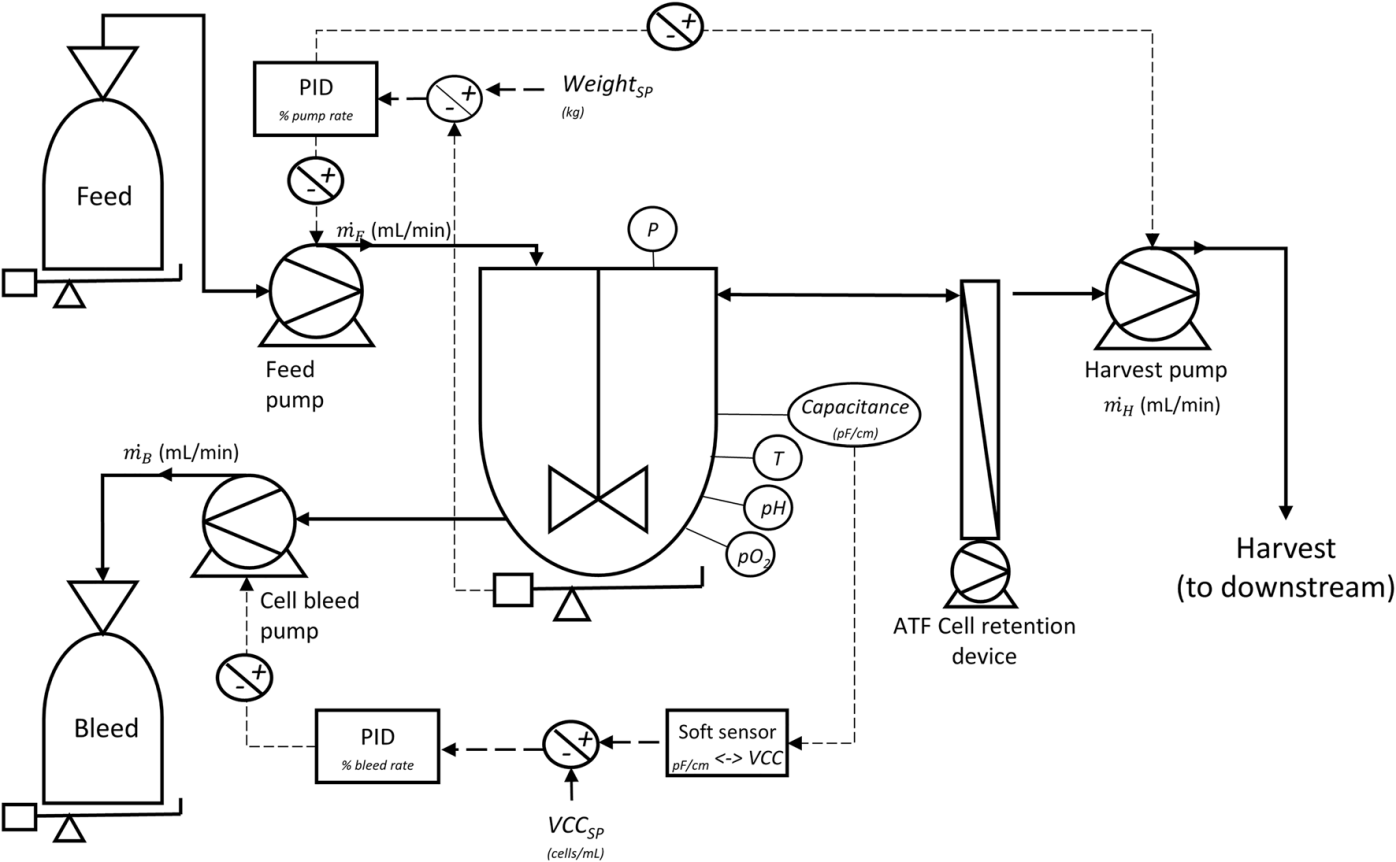
Schematic illustration of a state-of-the-art automated perfusion system with two feedback control loops for biomass and level control of the bioreactor

The term (*µ *− *B*) depicts the apparent growth rate and shall approach 0 under steady-state conditions in an optimal running perfusion process. A schematic representation of the perfusion process is shown in Fig. 1. The cells are remained in the bioreactor by a cell retention device, assuming 100% retention. After the initiation phase (batch mode), a continuous flow of feed and effluent is started, causing the cell concentration to approach its maximum. Equilibrium is established and cell and substrate concentrations become invariant. The culture is then regarded as having reached a steady state, as the key state variables no longer change. Hence, the boundary condition for the simulation which is performed under steady-state conditions is, therefore, defined as:

9 $$\frac{\mathrm{d}X}{\mathrm{d}t}=\frac{\mathrm{d}s}{\mathrm{d}t}=0.$$

When equilibrium is reached, we assume that the specific substrate consumption rate is proportional to the growth rate. Hence, if the same perfusion rate and feed concentration are applied, the culture will reach the same steady state. Therefore, the growth rate was modeled being independent of the availability of the substrate concentration, as the bleed control is only active in the steady-state phase when equilibrium is present. To mimic the influence of biological behavior, we applied large uncertainties of ± 20% to the cell-specific growth rates with respect to its nominal value, according to the probability density function by means of the python function numpy.random.normal(). The noise randomization was performed in each round of iterations.

The input parameters for the study were dependent on the control algorithm used (see “Monte-Carlo simulation” section). Product concentration and harvest flow rate were defined as output parameters since they can be considered as critical process parameters for subsequently connected downstream units. Simulations were performed using PyCharm 2021.3.2 (numpy, pandas, bokeh, scipy packages) by solving the differential mass balance equations.

### Implemented control strategies

To fulfill the objective of maintaining biomass in steady state in a perfusion process, two simple control strategies can be used. In control strategy A (Eq. 10), the perfusion rate was set constant and only increased when cells grew. The bleed rate is the adjusting parameter and mass balance was closed by the resulting variable, the harvest rate:

10 $$H=P\left(\mathrm{const}.\right)-{B}_{\left(\mu \right)}$$

In control strategy B (Eq. 11), the harvest flow rate is the constant parameter and bleed rate again adjusts according to cell growth. The perfusion rate is the resulting parameter and varies to fulfill the boundary condition.

11 $$P=H\left(\mathrm{const}.\right)-{B}_{\left(\mu \right)}$$

### Feedback and single prediction control

Two major control strategies have been applied to keep the biomass in steady state via regulation of the bleed pump as stated above. In feedback control algorithms, the objective function is to reduce the error signal to zero where

12 $$e\left(t\right)= {X}_{\mathrm{sp}}-{X}_{m}\left(t\right),$$

and *e*(*t*) is the error signal, *X*_sp_ the set point (SP) of the variable and *X*_*m*_(*t*) the measured value of the controlled variable or equivalent signal from the sensor. The feedback signal is transmitted via an online biomass sensor and accordingly the bleed pump is corrected to reach the target VCC (SP). It is assumed that the predicted biomass equals VCC, hence

13 $${X}_{m}\left(t\right)=\mathrm{VCC}=X.$$

The PUI is defined as the time difference between the measured value and the SP, representing the time interval for error signal calculation of the controller. To set the specific bleed rate in relation to the controller and the bleed pump, the bleed flow rate of the bleed is considered in the following control algorithm by:

14 $${b}_{m}= {V}_{r}\cdot B$$

Hence, the current VCC can be calculated as followed:

15 $${X}_{m}\left(t\right)={X}_{m}\left(t-1\right)-\frac{1}{{V}_{r}}*\Delta t*{b}_{m}\left(t-1\right)*{X}_{m}\left(t-1\right)*{e}^{\mu *\Delta t},$$

where *X*_*m*_(*t*) is the measured VCC at time point *t* and *X*_*m*_(*t *− 1) the previous VCC, *V*_*r*_ the bioreactor volume, *b*_*m*_(*t* − 1) the bleed rate during the last time interval, *µ* is the cell-specific growth rate and $\Delta t$ the time interval.

As a result, the calculated bleed rate (*b*_*m*_(*t*)) in the previous time interval [*t* − 1;*t*] (or corresponding PUI) is used for derivation of the predictive algorithm (Eqs. 17–19) and results in:

16 $${b}_{m}(t)= {(X}_{m}-{X}_{\mathrm{sp}})*\frac{\frac{V}{{X}_{m}}}{\mathrm{PUI}}+{b}_{m}(t-1)$$

Application of a predictive control algorithm on top of the feedback control signal (single prediction control) uses Eq. (15) in a similar way. A prediction value that would be reached if no future control action is taken is calculated as the difference value (*X*_diff_) between predicted value (*X*_*m*_(*t* + 1)) and target (*X*_sp_) within the prediction horizon *N*_p_, which is also defined as the length of optimization window:

17 $${X}_{\mathrm{diff}}\left(t+1\right)={X}_{m}\left(t+1\right)-{X}_{\mathrm{sp}}$$

In a next step, the controller solves the optimization problem by designing the best control parameter vector Δ*U*. In this way, the error function between the set point and predicted output can be minimized. The cost function *J,* therefore, is defined as:

18 $$J= {X}_{\mathrm{diff}}\left(t+1\right)+ \sum_{i=1}^{{N}_{\mathrm{c}}}{\Delta U\left(t\right)}^{\text{T}}*R*\Delta U\left(t\right)$$

where *X*_diff_(*t* + 1) is the predicted error, Δ*U*(*t*) is the optimal sequence of changes in the input, *R* is the design parameter and *N*_c_ is the control horizon. After fitting to this simulation study, the MPC has a prediction horizon of 12 h, a control horizon (PUI) of 6 h and the weighting in the optimization problem is *R* = 5.

The optimal solution is linked to set point signal *b*_m_ and to the state variable *X*_m_, where the predicted bleed rate (*b*_m_(*t* + 1) can be calculated until the next pump update interval as described in Eq. (19), allowing a correction of the value, if *b*_m_(*t* + 1) is not equal to *b*_m_(*t*). Hence, the bleed rate (*b*_m_) can be corrected to a minimized error signal before being transmitted to the controller:

19 $${b}_{\mathrm{m}}\left(t+1\right)=\frac{{X}_{\mathrm{diff}}\left(t+1\right)}{{N}_{\mathrm{p}}}*\frac{{V}_{r}}{{X}_{m}\left(t+1\right)},$$

20 $${{b}}_{{m}}\left({t}\right)={{b}}_{{m}}\left({t}+1\right).$$

The logic of the two different control algorithms are summed up in Table 1 and described in several steps.

**Table 1:** Logic for control actions and algorithms for feedback (FC) and single prediction control (SPC)

Step	Actions for FC	Actions for SPC
1	Calculate *X*_*m*_(*t*) = VCC from biomass soft sensor at time point *t*	
2	Calculate the error signal* e*(*t*) according to Eq. (12)	Calculate the bleed rate *b*_*m*_(*t*) in the previous time interval (PUI)
3	Take corrective action and send to controller	Perform optimization problem and minimize error function between set point and predicted output (Eqs. 17–19)
3	Operate for defined PUI	Calculate predicted bleed rate *b*_*m*_(*t* + 1) using Eq. (19)
4	Go back to step 1	Correct the bleed rate *b*_*m*_(*t*) if it is unequal to *b*_*m*_(*t* + 1) (Eq. 20)
5		Set the new bleed rate *b*_*m*_
6		Operate the process for the defined *N*_p_
7		Go back to step 1

### Monte-Carlo simulation

The simulations, statistical analysis and visualization were done in python 3.8 using Monte-Carlo simulations. The process time was defined up to 30-day steady state production. The Monte-Carlo simulation setup consists of several model parameters (see Table 2) for individual simulations that loop through different target set points in various iterations. The altered variables were taken partly from the literature or from empirical values. Process volume was defined being constant 10 L (*V*_*r*_ = constant), cell-specific productivities (*q*_p_) were set constant to 20 pg/cell/day, maximum of bleed rate (*B*_max_) was limited to 40% of the perfusion rate and the range of other variables is given in Table 2.

**Table 2:** Overview of iteration parameters in Monte-Carlo simulations

Model parameters	Set point		
Target biomass (*X*_SP_) (MVC/mL)	20	40	80
Cell-specific growth rate (*µ*) (1/d)	0.1	0.2	0.3
Perfusion rate (*P*) (L/L/d)	1	2	3

Note that the simulated specific growth rate deviates by ± 20% from its nominal value to illustrate large uncertainty in a biological environment. This is in good agreement with the behavior of steady state growth rates in experimentally performed perfusion processes (data not shown).

### Measures of skewness and kurtosis

The data set was further characterized of location and variability by including skewness and kurtosis. A data distribution is measured by symmetry by the value of skewness:

21 $${g}_{1}=\frac{\frac{\sum_{i=1}^{N}{\left({Y}_{i}-\overline{Y }\right)}^{3}}{N}}{{s}^{3}},$$

where *g*_1_ is referred to as the Fisher–Pearson coefficient of skewness, $\overline{Y }$ is the mean, s is the standard deviation and *N* is the number of data points. The skewness for a normal distribution is zero, therefore symmetric data should have a skewness near zero.

Kurtosis is a measure of whether data are heavy-tailed (presence of outliers) or light-tailed (paucity of outliers) relative to normal distribution. For univariate data, the formula for kurtosis therefore is:

22 $$\mathrm{kurtosis}= \frac{\sum_{i=1}^{N}\frac{{\left({Y}_{i}-\overline{Y }\right)}^{4}}{N}}{{s}^{4}},$$

where $\overline{Y }$ is the mean, *s* is the standard deviation and *N* is the number of data points.

## Results

### Design of control strategies

**Fig. 2:**
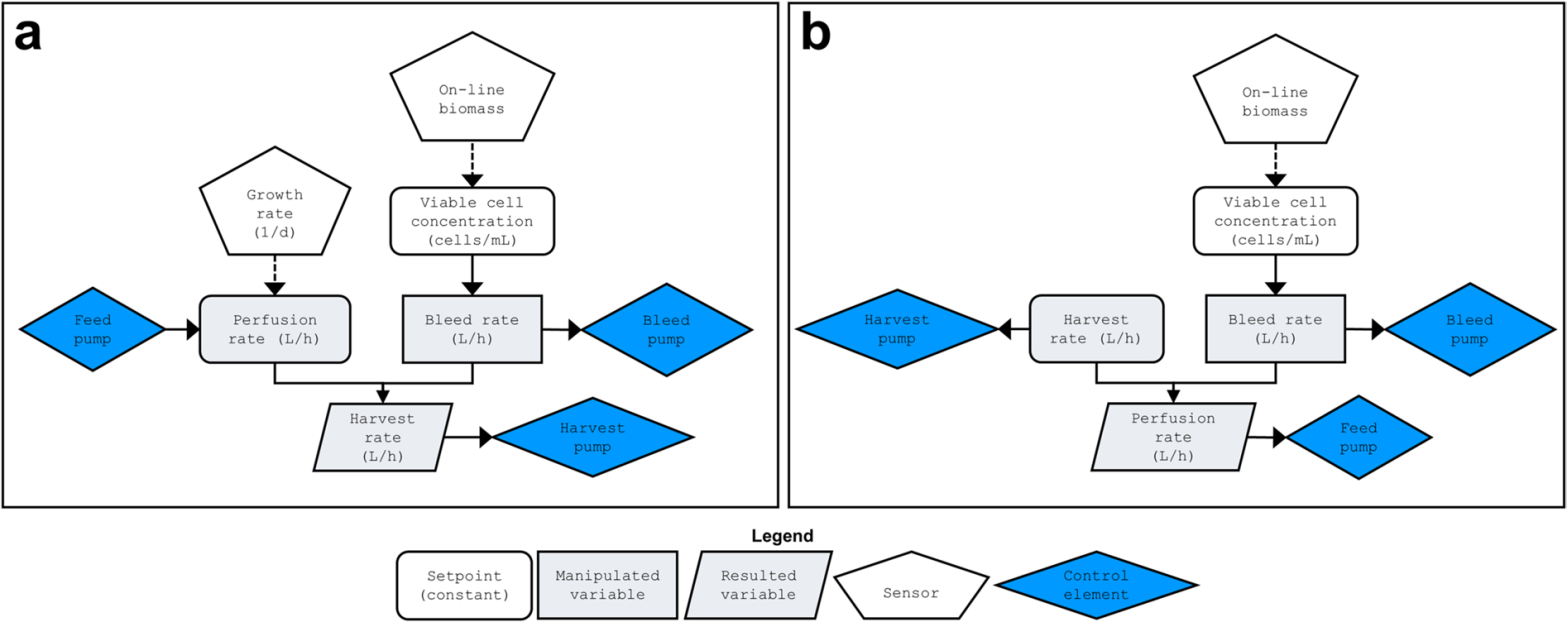
Flowchart overview of perfusion control strategies. (**a**) Control strategy A: constant perfusion rate, adapting bleed rate, and resulting harvest rate. (**b**) Control strategy B: constant harvest rate, adapting bleed rate, and resulting perfusion rate. Flowchart elements are described in the figure legend

The main objective of this study was to design and simulate perfusion control strategies that can be integrated to a continuous downstream unit. The simulations can be used to assess the degree of error propagation from the control algorithms of the upstream unit to the downstream unit, and to what extent it can be prevented or minimized. Two control strategies were designed in this study to investigate the different requirements for a single perfusion unit compared to an ICB. Strategy A was a simplified control strategy designed to control only one perfusion unit over 30 days by maintaining constant perfusion rate (Fig. 2a). The bleed rate is defined as a manipulated variable (MV) since it is controlled in a feedback loop based on the signal from an online biomass probe. The harvest rate is the inconstant variable (IV), responsible for closing the mass balance. Thus, the perfusion and bleed rate, as well as the growth rate and viable biomass, serve as input values for this model, and the harvest rate is the calculated output.

The alternative control strategy B was applied as an approach to control an integrated continuous bioprocess over the same time period (Fig. 2b). In strategy B, a defined harvest rate (SP) together with the manipulated bleed variable results in the output parameter of a flexible perfusion rate to fulfill the boundary conditions.

**Fig. 3:**
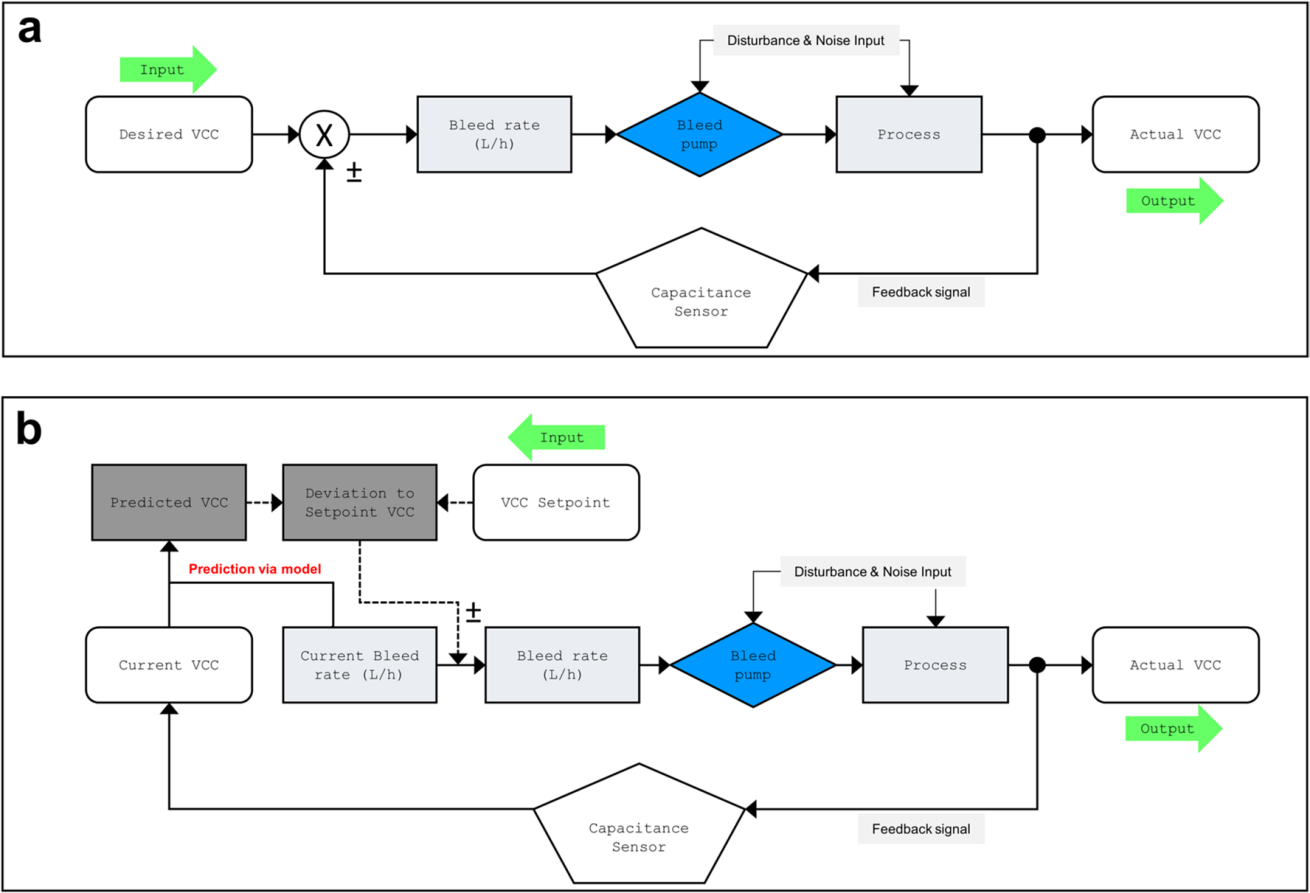
Flowcharts of feedback and model predictive controller. (**a**) Feedback controller (FC): control of biomass in the perfusion system via upregulation and downregulation of bleed rate. Feedback signal is derived from an online capacitance sensor. (**b**) Single prediction controller (SPC): model predictive controller as one-step-ahead control strategy for biomass in a perfusion system. A prediction model is applied as a control action on the error signal prior to its transmission to the controller

A state-of-the-art feedback controller (FC) was used to stably control the biomass in a closed-loop system (Fig. 3a). On the other hand, we applied a derived straightforward method that is based on feedback control, the so-called single prediction control (Fig. 3b). Notably, this method is always one-step ahead: a single predictive control algorithm (prediction model) is applied as a control action on the error signal prior to its transmission to the controller. In other words, the SPC observes the value that would be reached by the system output if no future control action is taken.

### Potential of SPC for VCC control

**Fig. 4:**
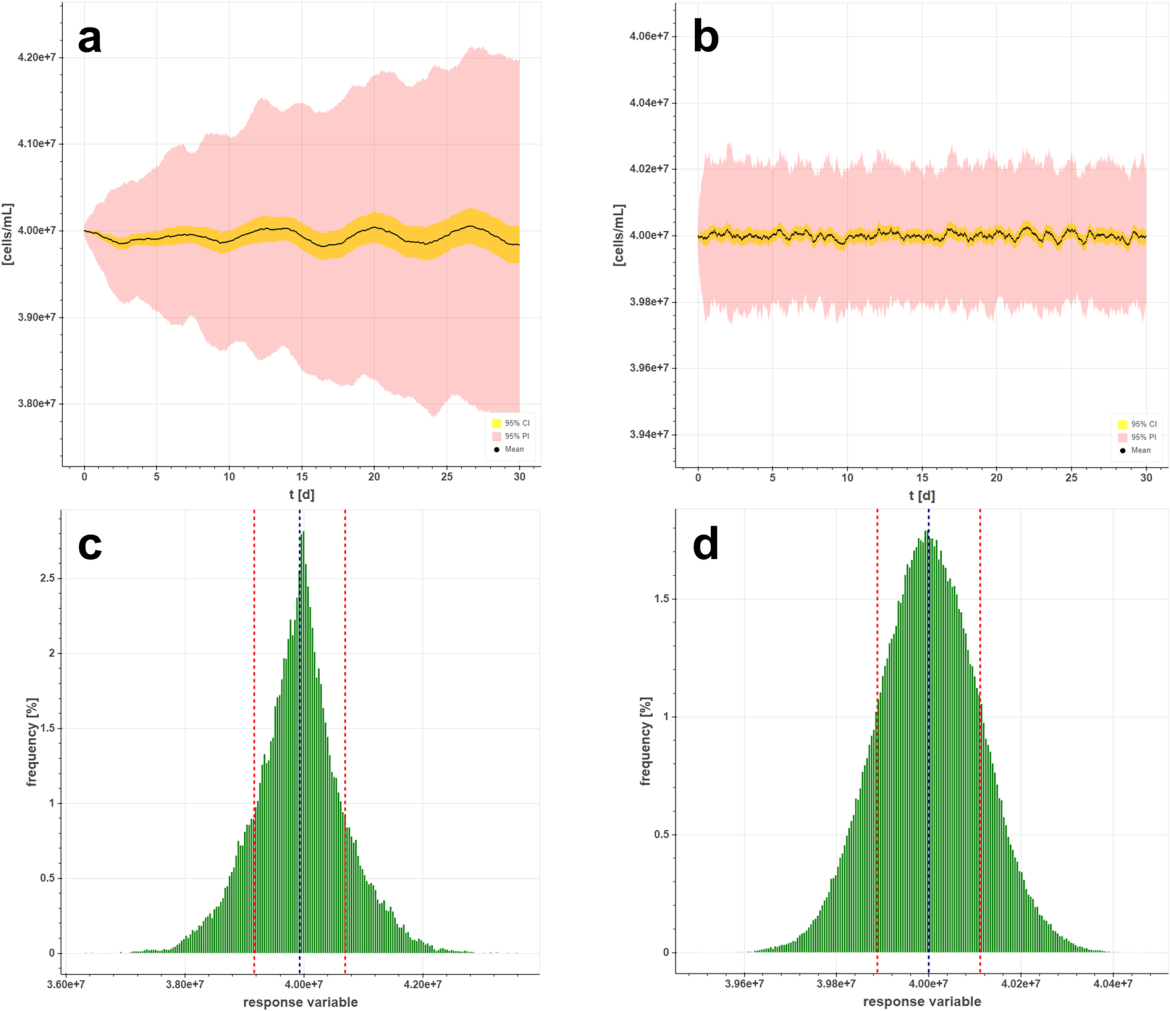
(**a**) Simulated VCC progression with feedback controller (FC) algorithm using strategy A. (**b**) Single prediction control (SPC) algorithm for calculation of VCC using strategy A. (**c**) Error distribution histogram of VCC response variable by FC. (**d)** Error distribution histogram of simulated VCC by SPC

The use of feedback controllers for controlling bioprocesses is state of the art, and is widely applied to fast reactions and processes [18, 20, 34]. We performed a simulation study with pump update intervals (PUI) of 6 h for feed, bleed and harvest pumps with both control strategies A and B. As an example, Fig. 4 shows the progression of VCC over a 30-day process time simulated with FC (Fig. 3a) and SPC (Fig. 3b). Models were calculated in a closed-loop control using strategy A (Eq. 10) for maintaining an SP of 40 MVC/mL in steady state at a cell-specific growth rate of *µ* = 0.2 1/d in a perfusion bioreactor with a 10 L working volume and perfusion rate of *P* = 2VVD. The mean VCC values were calculated over all iterations (*n* = 100). Additionally, confidence intervals of the main values (yellow area) and of future values (red area) were calculated with a probability of 95%. In Fig. 4a, the simulated VCC values show fluctuations with increasing amplitude when an FC is applied. The confidence and prediction band widen as the process progresses. Moreover, the deviation from the target value increases over time, with the maximum deviation in the VCC prediction being ± 0.2 MVC/mL.

The SPC uses the time interval of the PUI as control horizon *N*_c_ and a prediction horizon of 12 h to compute the corresponding future error. After solving the optimization problem and implementation of a design parameter to adjust transient behavior, the error function between set point and predicted output could be minimized. In Fig. 4b, the simulated VCC yields a very stable process compared to FC. We chose to use different figure scaling between FC and SPC to fully illustrate the precision of the method. The fluctuations of the VCC are negligible, with a maximum range of ± 0.02 MVC/mL. The confidence interval remains in same range throughout the entire process time. The same performance was obtained for higher time intervals (PUI of 24 h, 48 h, or 72 h) (see Appendix Fig. 9).

Error histograms were conducted to present the error distribution of the simulated data set from the feedback and predictive control strategies (Fig. 4). With the FC strategy, the SP of 40 MVC/mL did not coincide with the most frequent value (Fig. 4c). The center of the data was near the SP value, the distribution was symmetrical and the minimal and maximal values were between 37 and 43 MVC/mL. As shown in Fig. 4c, the distribution of deviations from SP did not follow a normal distribution curve. On the other hand, the SPC strategy resulted in a normally distributed deviation of the VCC values (Fig. 4d) from the SP. The distribution only deviated ± 0.03 MVC/mL from the target VCC. The most frequent value coincided with the target SP.

**Fig. 5:**
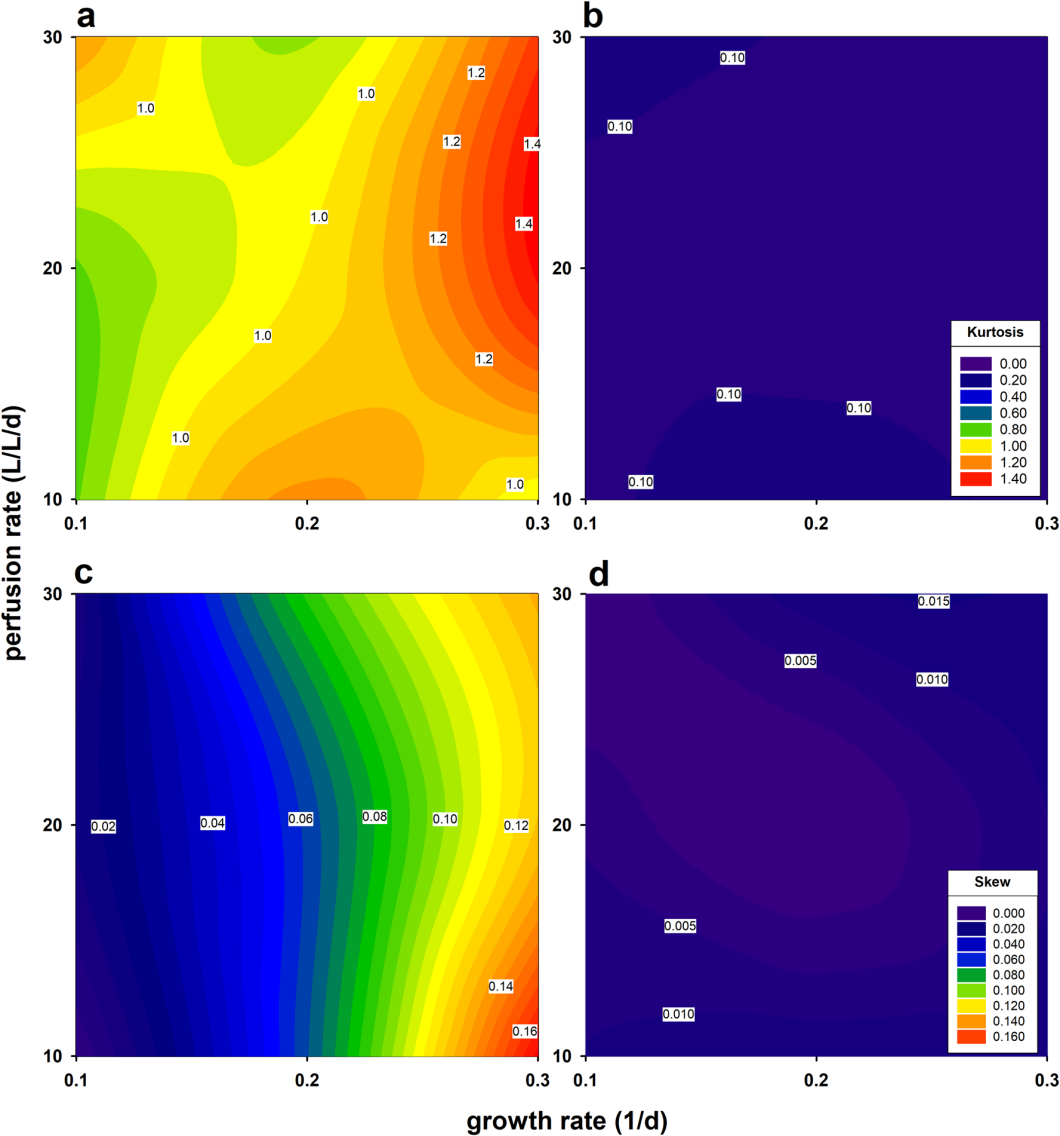
Contour plots of kurtosis and skewness variables related to perfusion and growth rates of simulated viable cell concentrations. Relationship between kurtosis for the feedback control FC (**a**) and (**b**) for the single prediction control SPC. Relationship between skewness for the FC (**c**) and (**d**) for the SPC

We set up a data set of 30 different perfusion runs, simulating combinations of various set points, including perfusion rates of 1–3 VVD, a target VCC of 20, up to 80 MVC/mL, and growth rates of 0.1–0.3 1/d. For all simulated FC conditions, we observed high skewness and kurtosis values of the distribution of the deviation from the SP (Fig. 5a, c). The contour plots for the FC strategy generally showed values greater than 0. The error increased with the growth rate. With the SPC, errors followed a normal distribution, were independent of the growth and perfusion rates, and scattered narrowly around 0 (Fig. 5b, d).

### Error propagation to downstream

**Fig. 6:**
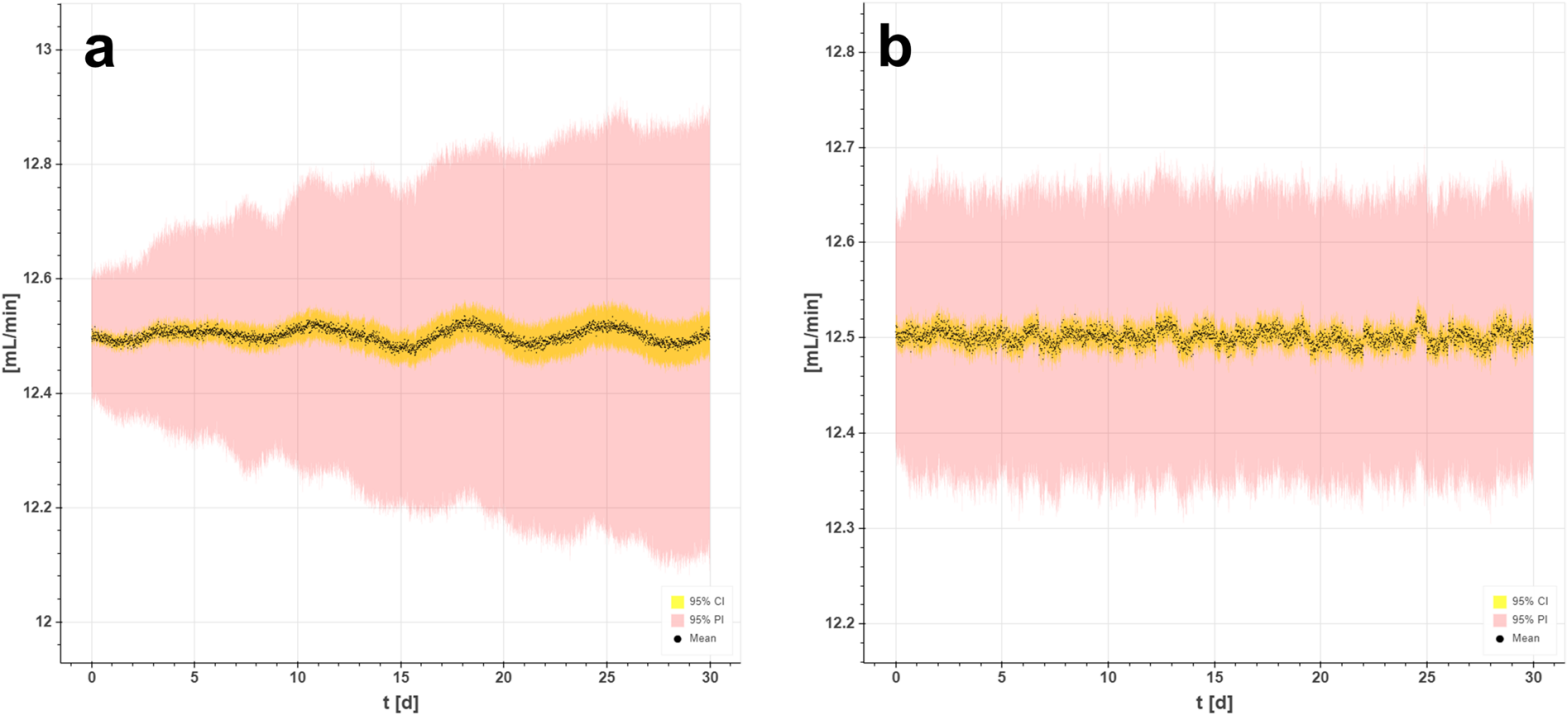
Simulated harvest flow rates in an integrated continuous bioprocess using (**a**) feedback and (**b**) single prediction control strategies

The product concentration (Appendix Fig. 8) and the flow of the harvest (Fig. 6) followed a similar trend as that observed for the VCC. With the FC, we observed a propagation of the error and fluctuation with increasing amplitude over time. With the SPC, both the product concentration in the harvest and the flow rates were stable, and the deviations from the set points were normally distributed. With the FC strategy, the error of biomass became increasingly large as the process progressed and the system lost its equilibrium, such that steady-state conditions were no longer 100% given. These fluctuations were observed to the same extent in the product concentration when the process was simulated with a constant *q*_p_ of 20 pg/cell/day and a normally distributed error of ± 20% (Appendix Fig. 8a). The maximum variations in product concentrations were ± 0.04 g/L. With the SPC (Appendix Fig. 8b), the product concentration could be kept constant (steady state) over a long process duration, and fluctuated in only very small amounts, with very narrow confidence and prediction intervals. Moreover, the maximum deviation of the harvest flow rate (± 0.35 mL/min) with traditional feedback control loops could be reduced by half (± 0.17 mL/min) by applying predictive control strategies (Fig. 6a, b).

### Product yield and losses with different control strategies

**Fig. 7:**
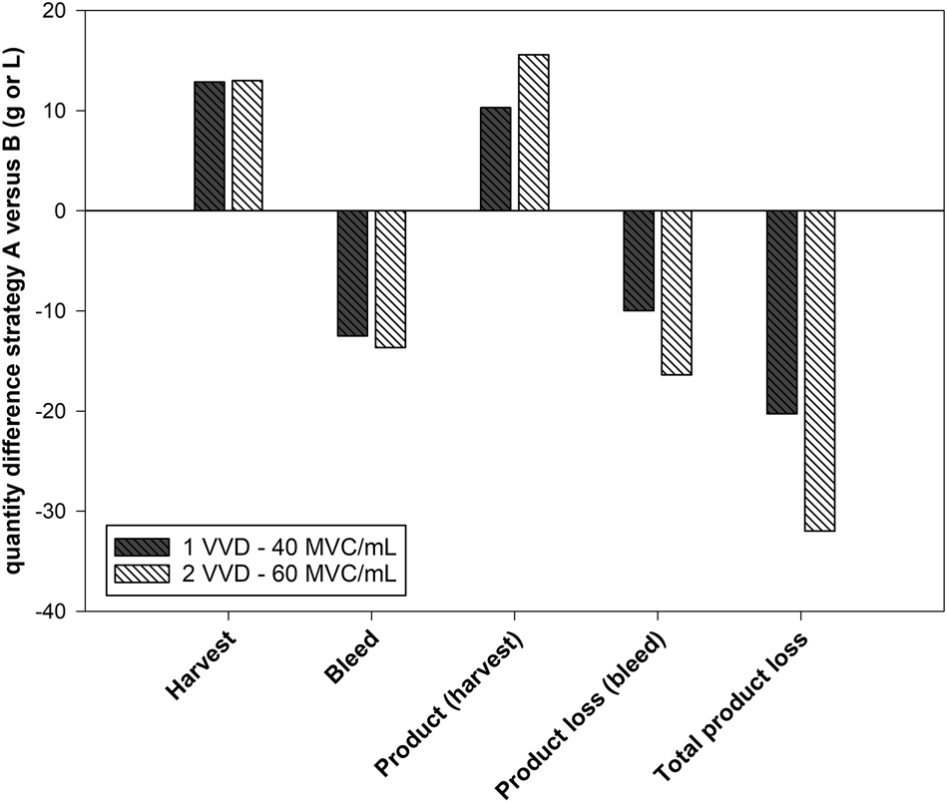
Quantity differences of control strategy A versus B. The total mass balances of total bleed, harvest volume, product yield and loss was calculated as difference between strategy A and B. Simulation was performed with two different perfusion rates and target cell concentrations (gray bar—1 VVD and 40 MVC/mL and white bar—2 VVD and 60 MVC/mL)

The differences and (dis)advantages of the control strategies of the perfusion unit (Fig. 2) in an integrated continuous process can be assessed in detail when the mass balance is closed over the entire process time. We simulated the differences in the total amounts of either harvest, bleed, or total product loss with strategy A compared to B, between 1 and 2 VVD and two different VCC set points (Fig. 7). When total feed was normalized, strategy B resulted in greater total product harvest, regardless of the biomass SP or perfusion rate compared to strategy A. Additionally, less total bleed was discarded. Conversely, this means that more product could be generated via harvest, but also that less product was lost in bleed when using strategy B over A. The total product loss was pronounced when using strategy A.

## Discussion

In a perfusion process, different control strategies can be applied to close the mass balance of input and output flow rates. In a basic strategy, the perfusion rate is kept constant (Eq. 10) and increased depending on the observed growth rate of the cells. In this scenario, the resulting variable is the harvest rate, which varies depending on the need to close the mass balance (Fig. 2a). However, this is not advantageous for controlling an integrated process, as there may arise many variations in the product stream from the biological deviation. Additionally, the resulting process will likely consume higher amounts of media (high cell-specific perfusion rates, CSPR). In contrast, we have presented an approach that meets the requirements of an ICB in which harvest flows are kept constant (Eq. 11) throughout the process (Fig. 2b). In this case, the perfusion rate is the varying parameter, which is changed to close the mass balance. This necessitates lower media consumption, as the perfusion rates are only increased when growth rate increases.

Particularly, the robust operation of units become more complex in an ICB when the first unit, the perfusion reactor, produces a highly fluctuating output with a tendency that the conditions derail with time. For example, the metabolism of animal cells is complex and can be influenced by various mechanisms of metabolite activation. Predictive control strategies can be used to better emulate this biological complexity [23, 35]. Besides the transient nature of animal cell culture bioprocesses, Zupke et al. also mentions interactions between CQAs and their control levers and delays associated with analytics as strong drivers for MPC [27]. Here, we implemented a single prediction control strategy (Fig. 3) for biomass control to overcome these issues. A stable set point control is possible without the need for time-delayed compensation due to noisy signals. For coping with long time delays in difficult process dynamics, predictive control has shown better performance compared to classical control algorithms [24–26, 35]. In this study, the control of biomass profoundly differed with FC and SPC strategies (Fig. 4). As shown in Fig. 4a, the FC caused oscillation of the SP value by constantly adjusting the bleed pump speed. The strategy attempted to compensate for the error through pump upregulation and downregulation, resulting in an error increase over time. This was particularly evident in the prediction interval, which widened with longer process times. In contrast, the SPC was capable of smoothly controlling the biomass in a continuous process by reducing the error differences between actual and target values (Fig. 4b).This control strategy combines the predictive capability with the classical use of feedback information, which improves the system performance in disturbance rejection. The predicted mean values were very close to the SP, and the same applied to the confidence and prediction intervals. The bands were very narrow to the mean, and showed no widening over time. This provided full steady-state behavior over 30 days of production, and the previously framed boundary conditions for an ICB were achieved. In addition, the model was confronted with larger prediction times. It has been shown that similar performance was achieved with longer pump update intervals (Appendix Fig. 9). Time delays and interactions can be compensated by already being one step ahead.

Furthermore, the model presented was evaluated by error distribution histograms (Fig. 4). The shape of the distribution of deviations provides an information regarding the quality of FC and SPC strategies. Only the error distribution in a SPC-controlled perfusion reactor was normally distributed, providing additional strong evidence that the FC is not optimally suited for controlling a perfusion reactor. The leptokurtic shape of the error distribution results from the increasing fluctuations of the difference between SP and process values (PV) over time. In addition, skewness and kurtosis results were considered in relation to perfusion and growth rates from a total of 30 different perfusion runs to identify combinations that result in low biomass prediction errors (Fig. 5). In the graphs, the valleys in the left corners (blue and green) represent growth and perfusion rate combinations that resulted in low prediction errors of VCC using FC (Fig. 5a, b). These widely distributed values were not seen in the contour plots of response values from model predictive control (Fig. 5c, d). Rather, all skewness and kurtosis values scattered very narrow around 0, as in a normal distribution. Neither growth nor perfusion rates seemed to influence the error distribution. With SPC biomass control, a stable process is always ensured, no matter how fast the cells grow or how high the pump rates are set.

In an integrated continuous process, it is important to also consider the output parameters (product concentration and harvest flow rates), as these can have an enormous impact on the connected downstream units. Due to the linear relationship between the specific product formation rate (*q*_p_) and biomass, the same picture emerges when the product concentration (g/L) is simulated (Appendix Fig. 8). The deviations propagate into the product stream via fluctuations in the cell concentrations. The difference of 0.04 g/L of product due to variations in the feedback control strategy would result in 5% lower product yield or 24 g less product for a 30-day ICB (*P* = 2VVD; 40 MVC/mL; *q*_p_ = 20 pg/cell/day).

If a strategy is chosen to regulate the perfusion process via the resulting harvest rates, an enormous challenge arises for the downstream capture step. In this case, the downstream unit must be able to react to fluctuations and errors to compensate for them. In Fig. 6a, the fluctuations in harvest flow are pronounced, and build up over a longer process duration. Steady state observance can no longer be assured. In contrast, with SPC, the process is in state of control, although the input and output flow rates adapt to cell growth and will change over time. However, as shown in Fig. 6b, when predictive control is applied, nearly the same harvest flow performance can be achieved as if a different control strategy (constant harvest flow rate) was applied by design (Fig. 2b).

Control strategy B addresses the opportunities and challenges of an ICB, with the aim of proposing an ideal control strategy. Simulation of a process with strategy B (*P* = 1 VVD; 40 MVC/mL) in Fig. 7 resulted in a total product loss of about 20 g and 32 g (*P* = 2 VVD; 60 MVC/mL) less compared to that with strategy A (total protein production of 240 and 700 g). The advantage is the perfusion rate as adjusted parameter according to cell growth, and the harvest rate is a fixed parameter independent of cell growth. In any case, this would lead to slightly less medium consumption, since the perfusion rate can be kept low if cell growth is slow (minimum CSPR).

Finally, Table 3 presents a global control strategy for a perfusion process directly connected to a downstream unit without mass flow interruption using multiple control stages. A constant perfusion rate (feedback control) has the advantage of being simple to implement, but requires prior knowledge of the relationship between the growth and perfusion rate of the culture. However, when the single prediction control strategy is applied to the bleed loop, stable and satisfactory control can be ensured for long process times.

**Table 3:** Proposed ideal global control strategy for a perfusion process integrated to downstream unit without mass flow interruptions

Number	Control loop	Measurement (online)	Input variable	Control algorithm	Manipulated variable
i	Bleed	Biomass sensor/Soft sensor	Viable cell density	MPC-Single prediction control (Eqs. 14–19)	Bleed rate setpoint
			VCC setpoint		
			Growth rate		
			Pump update interval		
ii	Bleed rate	Flow sensor or scale	Actual PV	Feedback control	Cell bleed rate
			Bleed rate setpoint		
iii	Feed rate	Flow sensor or scale	Actual PV	Feedback control	Perfusion rate
			Setpoint (const.)		
iv	Harvest		Cell bleed rate	Mass balance equation (Eq. 10)	Harvest rate setpoint
			Perfusion rate		
v	Harvest rate	Flow sensor or scale	Actual PV	Feedback control	Harvest rate
			Harvest rate setpoint		

Constant perfusion rate—MPC bleed control and resultant stable harvest flow with minimal fluctuations and disturbances

In this scenario, the harvest rate is less affected, as a resultant variable from the global mass balance. Therefore, a mass flow that does not fluctuate and is not subject to disturbances is transported to the downstream unit. This supports a general facilitation of the control regime, and is, thus, proposed as the optimal solution for the integration of the two units.

## Conclusion

The perfusion process is the most common way to continuously cultivate mammalian cells. This method enables cells to be continuously feed with medium, while they are retained in a bioreactor. Since it is not possible to fully arrest the growth of cells in the bioreactor, excess cells must be removed, also known as bleed. Proper control of the bleed rate is essential for a stable process. In an integrated continuous bioprocess, the harvest is directly processed further to downstream operations. For process integration, model predictive control is important, particularly when the capture step is sensitive to fluctuations in product concentration and product streams. Including a simple model—assuming a linear relationship between the bleed rate and growth rate of cells—can significantly improve the performance of the process. With the application of this algorithm, harvest flow and product titer do not fluctuate compared to with a conventional feedback controller. This will reduce control efforts in the subsequent units. The technology of using model predictive control in perfusion processes can be considered as a paradigm shift. With MPC, the potential of ICB can be fully exploited, since both product quality and process robustness can be directly controlled. Simply by adding a different control strategy, a process can also be made more economic with regards to better media utilization and reduced total product loss (via bleed). However, for robust and reliable next-generation processes, much work remains to be done, especially in the area of ICB conform automation and digitalization solutions.

## Figures and Tables

**Fig. 8:**
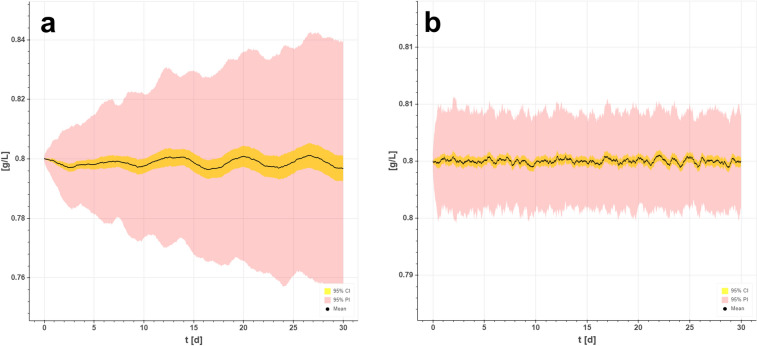
Simulated product concentration in an integrated continuous bioprocess using (**a**) feedback and (**b**) single prediction control strategies

**Fig. 9:**
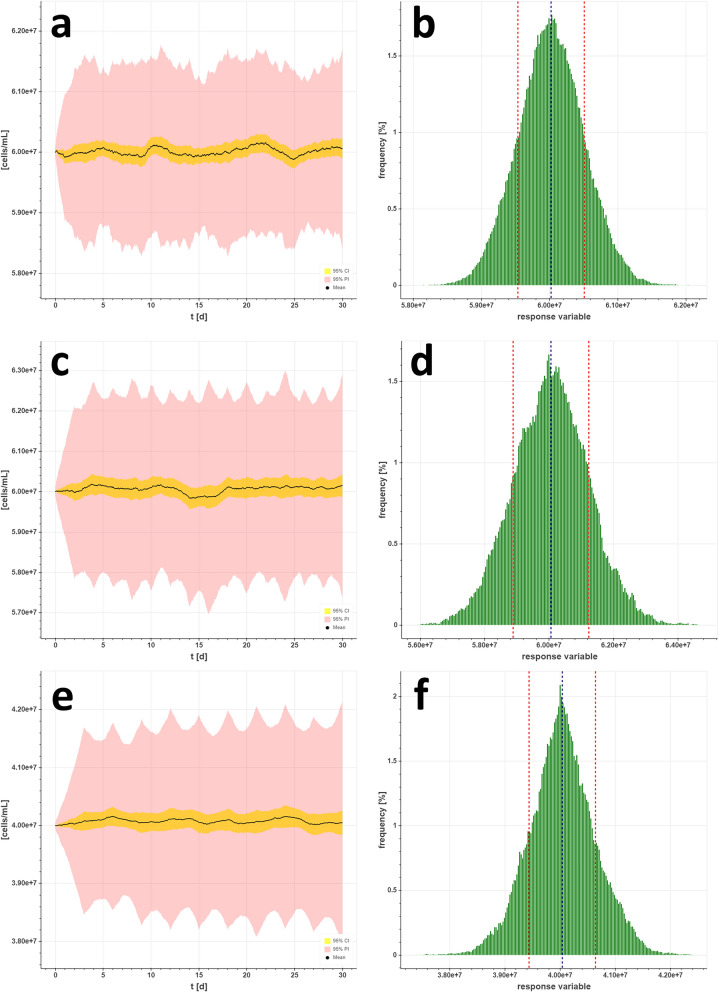
Simulated viable cell concentrations (VCC) and error distribution via single prediction control (SPC) using a pump update interval (PUI) of 24 h (**a**, **b**) and a PUI of 48 h (**c**, **d**) with parameters set to 60 MVC/mL, growth rate of 0.3 1/d and perfusion rate of 2 vessel volumes per day (VVD). Another simulation was performed with a PUI of 72 h (**e**, **f**) and with parameters set 40 MVC/mL, growth rate of 0.2 1/d and perfusion rate of 1 VVD

## Appendix

See Figs. 8, 9.

## Abbreviations

CSPR: Cell-specific perfusion rate;
ICB: Integrated continuous bioprocess
IV: Inconstant variable
MPC: Model predictive control
SPC: Single prediction control
FC: Feedback control
MV: Manipulated variable
MVC: Million viable cells
*N*_c_: Control horizon
*N*_p_: Prediction horizon
PAT: Process analytical technology
PUI: Pump update interval
PV: Process value
*q*_p_: Cell-specific productivity
SISO: Single input single output
SP: Set point
VCC: Viable cell concentration
VVD: Volume per volume per day
